# Appropriate Treatment Intensity for Diffuse Large B-Cell Lymphoma in the Older Population: A Review of the Literature

**DOI:** 10.3390/hematolrep16020032

**Published:** 2024-05-24

**Authors:** Satoshi Yamasaki

**Affiliations:** 1 Department of Hematology, St. Mary’s Hospital, 422 Tsubukuhonmachi, Kurume 830-8543, Japan; yamas009@gmail.com; Tel.: +81-942-35-3322; Fax: +81-9442-34-3115; 2 Department of Internal Medicine, Kyushu University Beppu Hospital, Beppu 874-0838, Japan; 3 Department of Hematology and Clinical Research Institute, National Hospital Organization Kyushu Medical Center, Fukuoka 810-0065, Japan

**Keywords:** older patients, diffuse large B-cell lymphoma, immunochemotherapy, comprehensive geriatric assessment, quality of life

## Abstract

Most patients with diffuse large B-cell lymphoma (DLBCL) are >65 years of age, with the number of patients expected to increase in the coming years. A comprehensive geriatric assessment that carefully evaluates fitness status and comorbidities is essential for selecting the appropriate treatment intensity. Although generally healthy patients or those <80 years of age may benefit from standard immunochemotherapy, unfit/frail patients or patients >80 years old may require reduced-intensity chemotherapy or less-toxic drugs. Some new drugs are currently being tested as single or combined agents for first-line treatment, aiming to improve the outcomes of conventional chemotherapy. This review systematically collates and discusses the outcomes associated with the use of immunochemotherapy in older patients with DLBCL, as well as considering the impact of full-dose immunochemotherapy on quality of life in older and frail patients, summarizing the rationale for reduced dosing in the older population, and presenting recommendations for selecting patients likely to benefit from reduced dosing. If preliminary efficacy and safety data are confirmed in future clinical trials, non-chemotherapy-based immunotherapy approaches could become an alternative potentially curative option in frail patients and those >80 years of age with DLBCL.

## 1. Introduction

Common comorbidities and limited organ reserves (e.g., bone marrow, heart, kidneys, liver) in older patients lead to common problems in treating older patients and place them at higher risk of toxicity compared with younger patients. Clinicians should also consider issues related to impairments in physical and cognitive function, which might compromise their ability to attend hospital or manage treatment at home, especially in the absence of a caregiver. In addition to factors related to patient fitness, unfavorable biological properties of the disease may also contribute to a poor prognosis. This section introduces the definition and epidemiology of diffuse large B-cell lymphoma (DLBCL) and highlights its increasing frequency and incidence, especially in older people, as well as presenting the characteristics of older patients (comorbidities, frailty, and decreased organ reserve) that might complicate management decisions and reduce tolerance to full-dose chemotherapy.

## 2. Chemotherapy Options

The aim of first-line treatment in fit patients ≤80 years old should be curative, with a total-dose anthracycline-based regimen. R-CHOP (rituximab, cyclophosphamide, doxorubicin, vincristine, prednisone) has been used as an initial treatment for over 20 years [1], and most attempts to improve its results by adding new biological agents to immunochemotherapy, especially in patients with non-germinal center B (GCB) DLBCL, have failed to show any significant advantage [2]. The PHOENIX trial found no improvement in event-free survival by adding ibrutinib to R-CHOP in patients with newly diagnosed non-GCB DLBCL, and ibrutinib plus R-CHOP was associated with increased toxicity in patients >60 years old, leading to compromised R-CHOP administration and worse outcomes [3]. No improvement was observed in the ROBUST trial in previously untreated patients with activated B-cell-type DLBCL receiving lenalidomide plus R-CHOP [4]. The POLARIX study is the only randomized phase III trial that has demonstrated significantly improved progression-free survival (PFS) in patients with DLBCL [5]. In this trial, replacement of vincristine with polatuzumab vedotin (Pola), an antibody-drug-conjugate targeting CD79b, in the R-CHOP scheme (Pola-R-CHP) improved the 2-year PFS to 76.7% compared with 70.2% for standard R-CHOP in patients aged 18–80 years with intermediate-risk or high-risk DLBCL, with similar safety profiles; however, the 2-year overall survival (OS) rates did not differ between the groups (88.7% in the Pola-R-CHP group and 88.6% in the R-CHOP group). Exploratory subgroup analysis revealed greater benefit in patients aged 60 years and older and in patients with non-GCB types, double expression, and high international prognostic index (3–5). The modest PFS benefit of Pola-R-CHP and similar OS has led to some concerns about the broad application of this regimen. Although Pola-R-CHP is a good choice, especially in certain patient subgroups, R-CHOP is likely to remain the standard of care in future clinical trials and an effective backbone for new combinations.

Dose-adjusted R-EPOCH (rituximab, etoposide, prednisone, vincristine, cyclophosphamide, doxorubicin) is one of the most suitable therapies for patients with high-grade B-cell lymphoma (double or triple hit). This intensive treatment, however, may not be acceptable for patients >80 years old and those <80 years with significant comorbidities [6,7], and there were no advantages of dose-adjusted R-EPOCH in patients >65 years with double/triple expression [8].

Although the cells of origin, molecular features, genetic features/expression (such as MYC, BCL-2, BCL-6, TP53, CDKN2A, and primary histocompatibility class II), and positron emission tomography-directed treatment approaches may contribute to the overall prognosis [9,10,11,12], there are currently few systems or treatment guidelines for patients aged >65 years, including in the European Society for Medical Oncology guidelines [13] and National Comprehensive Cancer Network guidelines [14], and R-CHOP remains an effective and appropriate therapeutic option for older patients with DLBCL [15].

## 3. Considerations in the Older Population

Aging is a complex natural process leading to progressive loss of physiological integrity, organ dysfunction, increased inflammation, and susceptibility to genetic damage and epigenetic modification. Some patients do not receive standard-dose chemotherapy because of their own preference or that of their physician, and the lack of this treatment may thus not be due strictly to patient age or comorbidities, leading to potential bias in terms of the relationship between older age and poor outcome risks, as previously noted [16]. There may thus be selection bias regarding the ineligibility for standard-dose chemotherapy because no proper randomization could be achieved. R-CHOP is not standardized for older patients with DLBCL, particularly for those aged ≥80 years [17,18].

Several characteristics of older patients may impact their ability to tolerate standard curative therapy, including comorbidities and organ reserve, the impact of age on pharmacokinetics (therapeutic index, reduced drug metabolism, and elimination), concomitant medication, and nutritional status/frailty [17,19,20]. Older patients at high risk, as defined by higher international prognostic index scores, low albumin, and Cumulative Illness Rating Scale for Geriatrics score ≥6, may be at higher risk of infection-related morbidity and mortality [21]. Some reports have also suggested differences in disease characteristics between younger and older patients, including dominance of the activated B-cell subtype [19,22]. There is also a distinction between poor clinical outcomes, such as lower OS, PFS, and complete remission (CR) rates (CRRs), and quality-of-life (QoL) outcomes, including capacity for daily activity, social functioning, and patients’ general health perception, with QoL factors potentially influencing individual preferences independent of clinical outcomes.

In addition, sarcopenia is a crucial factor affecting the physical fitness of the older population. Skeletal muscle index was recently reported to be the most appropriate assessment method for evaluating sarcopenia and a critical prognostic factor in terms of OS and PFS in older patients with DLBCL [23].

## 4. Management of Frail, Older Patients

Although outcomes differ in older compared with younger patients with DLBCL, OS may be improved with appropriate treatment [24], and age alone should not be a contraindication to chemotherapy [17,25]. Although reduced-intensity chemotherapy is associated with poorer outcomes in the general population, reduced dosing intensity does not consistently affect survival in patients aged >80 years compared with full-dose chemotherapy [26]. Although OS following attenuated-dose R-CHOP (R-mini-CHOP) is inferior compared with that of R-CHOP [27], R-mini-CHOP is being increasingly used as an initial treatment in the older population. Eligibility criteria other than age should thus be carefully considered when choosing a treatment for unfit/frail patients or patients >80 years old. A list of first-line immunochemotherapy regimens suitable for older patients with DLBCL is shown in Table 1.

## 5. Reduced-Intensity Chemotherapy

Unfit and frail patients usually require an adapted therapeutic regimen. R-miniCHOP is the most popular treatment for DLBCL in patients aged >80 years without severe comorbidities, with a 2-year PFS of 47% and a 2-year OS of 59% [28]. The LYSA group attempted to improve the outcomes of R-mini-CHOP by using ofatumumab instead of rituximab [29], but the 2-year OS (64.7%) was only slightly better. Nevertheless, this study confirmed the importance of a systematic pre-stage using prednisone and vincristine before immunochemotherapy, which could improve performance status and reduce treatment-related mortality in the first cycle [30]. Based on the promising results of the POLARIX trial, the Nordic Lymphoma Group is currently randomizing patients aged ≥80 years or frail patients aged ≥75 years to R-mini-CHOP or R-mini-CHP plus polatuzumab vedotin in phase III trials (ClinicalTrials.gov Identifier: NCT04332822).

Many older patients have cardiac comorbidities and multiple cardiovascular risk factors (e.g., hypertension, diabetes mellitus, chronic kidney disease), which are associated with an increased risk of anthracycline-related cardiotoxicity [16,31]. In these cases, replacing traditional doxorubicin with a non-pegylated liposomal form may reduce the risk of cardiac events without compromising efficacy [32,33,34,35].

For patients with a complete contraindication to anthracyclines, the European Society for Medical Oncology (ESMO) guidelines suggest that substituting doxorubicin with gemcitabine or etoposide [13], and omitting doxorubicin altogether (e.g., the R-CVP regimen), may be an option in frail older patients; however, it is generally less effective, and this option has a palliative purpose [36]. Tucci et al. recently reported that including rituximab within palliation improved the outcome in terms of 2-year OS (42% with rituximab vs. 22% without rituximab; *p* = 0.008) [37].

The safety and efficacy of rituximab and bendamustine in indolent lymphoma has prompted their evaluation as a first-line treatment in frail older patients with DLBCL, with an overall response rate (ORR) of 62% (53% CRR) in a phase II trial of 49 patients with DLBCL aged >70 years with significant comorbidities and declining health; however, the PFS was disappointing (38% at 2 years) [38]. The phase II ReRi study tested the combination of rituximab plus lenalidomide in 68 patients with newly diagnosed DLBCL who were not eligible for conventional cytotoxic therapy. The ORR was 41%, and the 12-month PFS and OS were 55% and 69%, respectively. Although the initial endpoint was not confirmed in this study, activity was observed in many cases, justifying its further exploration as the backbone of new chemotherapy-free combination therapy in older and frail patients [39].

Park et al. concluded that clinical outcomes following reduced-intensity R-CHOP chemotherapy were comparable to those of standard-dose R-CHOP in patients with DLBCL aged ≥65 years [20]. Eyre et al. examined a cohort of patients across eight centers in the United Kingdom and concluded that R-mini-CHOP resulted in similar OS, PFS, and relapse rates to full-dose R-CHOP in patients aged ≥80 years [40]. Peyrade et al. evaluated and recommended R-mini-CHOP as standard therapy in patients aged >80 years with DLBCL [28]. Liao et al. retrospectively compared R-CHOP, R-mini-CHOP, and R-CVOP in patients aged ≥75 years and concluded that OS was comparable and differences in PFS were insignificant [18]. Verner et al. undertook a phase 2 study of R-mini-CHOP plus ibrutinib in patients aged >75 years and showed that, although 2-year OS was 68%, ibrutinib was discontinued in 25% of patients, primarily because of adverse events [41].

Regimens other than R-CHOP that may be used at lower dose intensities have been introduced to treat frail older patients with DLBCL. Peyrade et al. demonstrated a 2-year OS of 64.7% in patients aged >80 years with DLBCL treated with ofatumumab, pre-phase treatment (oral vincristine followed by oral prednisone), and reduced-intensity CHOP [29]. Pola-R-miniCHP [5,42], as well as other chemotherapy options, including R-mini-CEOP, R-split CHOP, R-CVOP, and R-GCVP [18,43], may also be promising therapies for older patients with DLBCL.

## 6. New, Novel, and Chemotherapy-Free Regimens

A limited number of older patients with R/R DLBCL receive the traditional standard approach based on salvage chemotherapy (usually a platinum-containing regimen), followed by high-dose therapy and autologous stem cell transplantation (ASCT) in chemosensitive cases, ideally in patients with CR at re-staging with positron emission tomography/computed tomography. The upper age limit in most studies was 60 or 65 years [44,45], and only a few retrospective series, subgroup post hoc analyses, and data from international registries have described the outcomes of older patients [46,47,48]. In general, ASCT appears to be a feasible option in selected fit older patients ≤75 years of age, but the 1-year non-relapsed mortality in older patients was only 6.0%, suggesting that age should not be the sole factor determining a patient’s eligibility for ASCT [49]. Transplant-ineligible but chimeric antigen receptor (CAR) T-eligible older patients could soon become an accurate and relevant population [50]. Despite significant efficacy, many issues can limit the widespread application of CAR T-cells in clinical practice, particularly in older patients, including the need for specialized centers that may be far from the patient’s residence, a long turnaround time from leukapheresis to product release, and the cost of the entire treatment [51]. Evidence from randomized trials supports the use of a comprehensive geriatric assessment (CGA) to reduce treatment-related toxicities and guide treatment intensity in the care for solid tumors, while its use for evaluation of cellular therapies is less evidence-based.

The combination of tafasitamab–lenalidomide represents another possible salvage option for patients with DLBCL who are not eligible for transplant or CAR T-cells. Although tafasitamab, a new anti-CD19 antibody with an enhanced Fc portion, and lenalidomide have limited single-agent activity, in vitro and in vivo studies showed synergistic effects with limited toxicity [52]. The phase II single-arm L-MIND study enrolled 81 patients with DLBCL who had relapsed after one to three prior systemic regimens, including a minority of primary refractory cases [53]. Tafasitamab was administered in combination with lenalidomide for 12 cycles, followed by tafasitamab monotherapy until progression or toxicity. Updated analysis after ≥35 months of follow-up showed an ORR of 57.5%, including CR in 40% of cases, a median OS of 33.5 months, and a median PFS of 11.6 months. Tafasitamab–lenalidomide thus appears to allow long-term responses with a well-tolerated immuno-modulatory combination [54].

CD19 antigen is the target of loncastuximab tesirine, a novel antibody–drug conjugate that delivers a pyrrolobenzodiazepine dimer after binding to the B-cell surface and entering the cell [55]. The phase II LOTIS-2 study enrolled 145 patients with relapsed or refractory (R/R) DLBCL who had received at least two lines of therapy, including 20% of patients with primary refractory disease, 20% with transformed lymphoma, and 10% with double or triple hit lymphoma. The ORR in this study was 48.3%, with CR occurring in 24.1% of cases and a median duration of response of 10.3 months. The safety profile was acceptable, with the most relevant adverse events being neutropenia, thrombocytopenia, increased gamma-glutamyl transferase, and pleural effusions [56].

New drugs have recently emerged that may provide more effective treatment. Polatuzumab vedotin is a novel antibody–drug conjugate that delivers the microtubule inhibitor monomethyl auristatin E (MMAE) to B-cells and targets the CD79b antigen [57]. In a phase II study, 80 patients with R/R transplant-ineligible DLBCL were randomly assigned to receive Pola-BR (polatuzumab vedotin, bendamustine, rituximab) combination or BR alone [58]. The median number of prior treatment lines was two (range 1–7), and most patients (75–85%) were refractory to their last treatment. Pola-BR resulted in a significantly higher CRR (40.0% vs. 17.5%), longer PFS (9.5 vs. 3.7 months; *p* < 0.001), and longer OS (12.4 vs. 4.7 months; *p* = 0.002) compared with BR alone; however, Pola-BR patients had a higher rate of hematological toxicity but similar grade 3–4 infections. Peripheral neuropathy, usually associated with MMAE, was grade 1–2 in all cases and resolved in most patients. Updated outcomes from the randomized arm with a median follow-up of 48 months and the results for an extension cohort of 106 additional patients that received Pola-BR showed no new safety signals and confirmed a significant survival benefit [59].

Chemotherapy-free regimens, targeted therapies such as Bruton’s tyrosine kinase inhibitors, immunotherapies such as lenalidomide, and bispecific antibodies are novel options that may offer promising strategies in older patients (Table 2) [60,61,62]. The safety and effectiveness of chemotherapy-free strategies, such as ibrutinib, rituximab, and lenalidomide, have been tested as first-line options in frail or unfit patients aged ≥75 years [63]. The treatment landscape for B-cell lymphoma has recently been expanded by bispecific anti-CD20xCD3 antibodies that can engage and redirect patients’ T-cells to eliminate malignant B-cells [64,65,66,67]. The main advantage of this approach is its ready-made, rapid availability and toxicity similar but generally inferior to CAR T-cells. Glofitamab, a bispecific antibody characterized by a novel 2:1 CD20–CD3 binding configuration, showed a high response rate (ORR 52%, CRR 39%) in 154 patients (median age 66 years) with R/R DLBCL after treatment, who had received at least two prior treatments, and 52 had already received CAR T-cell therapy. CR continued at 12 months in 78% of cases, and the most frequent adverse event expected of this class of drugs was cytokine-release syndrome (all grades 63% of patients, grade ≥3 4%) [64]. Epcoritamab is a good choice for older patients because of its subcutaneous mode of administration. The phase I/II dose-expansion cohort included 157 patients with DLBCL (median age 64 years), who showed an ORR of 63%, a CRR of 39%, and a median duration of response of 12 months with continued treatment. Cytokine-release syndrome occurred frequently but was only grade 1–2 in most cases (97%) [65]. These novel and chemotherapy-free options may thus be valid first-line therapy choices for older/frail patients and may play a more significant role in older relapsed patients, including as second-line and third-line therapies.

## 7. Geriatric and QoL Assessments, and Tailoring Therapy in DLBCL

CGA has been used to help identify older patients with lymphoma suitable for standard-dose chemotherapy [71]. The American Society of Clinical Oncology expert panel suggested that clinicians should consider CGA for older chemotherapy patients [72]; however, few reports have considered the CGA as an effective tool for guiding therapeutic strategies in patients aged ≥80 years with lymphoma who received reduced-intensity chemotherapy [73].

Treating older patients with DLBCL presents the clinical dilemma of balancing the potential for cure while minimizing toxicity. Although age itself is not a contraindication to full-dose curative therapy, comorbidities or functional impairments may suggest the need for dose reduction or drug substitution to improve tolerance. There is wide heterogeneity among older patients, and traditional measures of performance status are not accurate enough to define treatment goals and adjust treatment intensity. ESMO guidelines recommend the application of a geriatric assessment to avoid the risk of under- or over-treatment [74]. The Fondazione Italiana Linfomi recently conducted a simplified geriatric assessment (sGA) in a large prospective series of patients with DLBCL aged >64 years (the Elderly Project), based on age (≥ or <80 years), Cumulative Illness Rating Scale for Geriatrics (CIRS-G), activities of daily living, and instrumental activities of daily living (IADL) [75]. The sGA is an objective and reproducible tool that can be easily administered by a single hematologist (in <10 min) and permits the classification of older patients as fit (54%), unfit (28%), or frail (18%), with significantly different outcomes.

Choosing between these different options may be difficult in clinical practice, and real-world data report poorer outcomes than those seen in clinical trials, likely due to the smaller number of patients selected [76,77]. In the absence of randomized trials, comparisons between drugs are only derived from retrospectively matched cohorts, and the results should thus be interpreted cautiously. The main factors affecting treatment decisions are the aim of the therapy (CR, duration of response, QoL), patient characteristics (age, fitness, comorbidities), logistic and social aspects (presence of caregiver, distance from hospital), and disease characteristics (previous treatment line, refractoriness).

The assessment options, supporting evidence demonstrating the relative complexity of the assessment tools, and their crucial evaluation components are summarized in Table 3. The relative complexity of a CGA necessitates the use of sGAs and evidence for the use of components of the CGA (e.g., IADL [78]) [79] and the development of screening tests (e.g., G8 [80]). Additional clinical criteria that may aid therapeutic decision making include cell subtypes such as GCB-cell-like and non-GCB subtypes [81] and lymphocyte/monocyte ratio [82].

Clinical prognosis alone might be insufficient to individualize therapy, and significant improvements in daily activity and social attitude scores (QOL-ACD) and general health perception and social functioning (SF-36) in terms of QoL have been demonstrated in lymphoma patients aged ≥80 years [86]. No other studies, however, have investigated improvements in QoL in relation to reduced-intensity chemotherapy.

In the future, it may be necessary to consider treatment strategies that emphasize QoL from the perspective of patient communication and consent to treatment, in parallel with or in addition to the known clinical benefits of chemotherapy for DLBCL.

**Figure 1:**
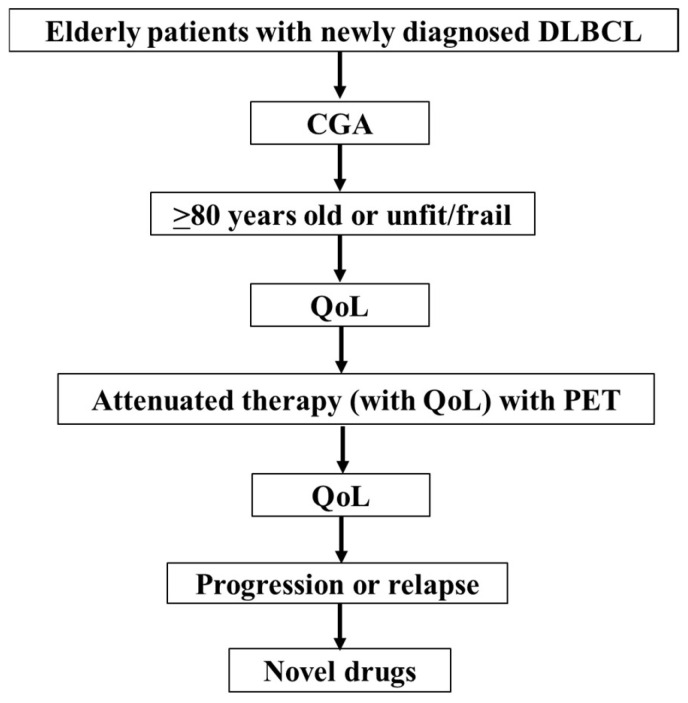
My approach to older patients with DLBCL. DLBCL, diffuse large B-cell lymphoma; CGA, comprehensive geriatric assessment; QoL, quality of life; PET, positron emission tomography.

**Figure 2:**
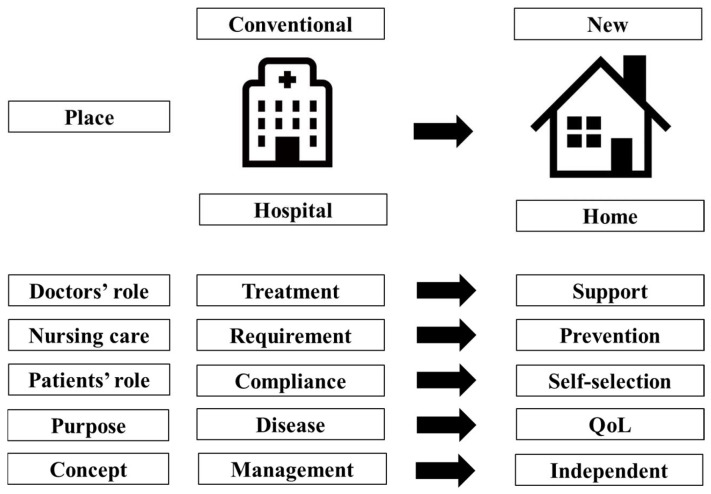
Paradigm shift in lymphoma treatment in the older population. QoL, quality of life.

There is currently no standard therapy for older patients with DLBCL, particularly those aged >80 years, and the treatment choice is based on a combination of information, such as G8, IADL, Charlson comorbidity index, daily life, and cognitive status [87] supporting immunochemotherapy, alternatives to chemotherapy, impact on QoL, and evaluation of frailty/fitness. There is a need to proactively consider critical features that will help select appropriate patients for alternatives to standard treatments, and the roles of formal or informal pre- and post-treatment QoL assessments in guiding practice. Careful administration of attenuated immunochemotherapy was shown to improve clinical outcomes and QoL in clinical practice, compared with no treatment, and CGA, abbreviated frailty assessment, and improvement in QoL were observed in a small number of cases [88]. Performance of standardized, systematic CGA by geriatricians permits older patients with lymphoma to be classified according to frailty, with significant differences in terms of clinical outcomes across groups [89]. I present my clinical approach for older patients with DLBCL (Figure 1) and a paradigm shift (from management to independent) in lymphoma treatment in the older population (Figure 2). It is expected that the involvement of multiple individuals in multiple areas, such as nursing care (from requirement to prevention), purpose of treatment (from disease to QoL), patient’s role (from compliance to self-selection), doctors’ role (from treatment to support), and place (from hospital to home) may play an important role when selecting less intensive immunochemotherapy for frail patients, especially those >80 years of age.

## 8. Future Directions and Conclusions

As noted above, many efforts have been made to improve the standard R-CHOP regimen by adding new targeted drugs (so-called R-CHOP plus X trials), but these still need to demonstrate better OS. Future trials will attempt to achieve better outcomes by combining R-CHOP with new Bruton’s tyrosine kinase inhibitors characterized by a more favorable profile, such as zanubrutinib (ClinicalTrials.gov Identifier: NCT05189197). In addition, the front-MIND phase III trial (ClinicalTrials.gov Identifier: NCT04824092) has tested the efficacy and safety of the combination of tafasitamab–lenalidomide and R-CHOP in patients aged 18–80 years with newly diagnosed DLBCL, with high/intermediate or high-risk disease.

Investigators at the MD Anderson Cancer Center have proposed a different strategy, based on an initial phase with biological agents alone (RLI: rituximab, lenalidomide, and ibrutinib) administered for two cycles to patients with non-GCB DLBCL, followed by the addition of conventional chemotherapy (either R-CHOP or R-EPOCH). Despite the small number of patients (60 in total, of which 28% ≥70 years), this “Smart Start study” paves the way for targeted therapy before chemotherapy, showing an impressive response rate after RLI alone (ORR 86%, CRR 36%), with a final ORR of 100% and a 2-year PFS of 91% [90].

Chemotherapy-free treatments based on new antibodies and small molecules represent an emerging approach for unfit/frail older patients who are not eligible for standard chemotherapy. A phase I/II study explored the use of the bispecific antibody mosunetuzumab as first-line treatment in patients with DLBCL aged >80 years, or aged >60 years with comorbidities precluding full-dose chemoimmunotherapy. The ORR and CRR were 56% and 43%, respectively, in 54 patients with a median age of 83, with no grade ≥3 cytokine-release syndrome and no neurotoxicity [91]. An incoming phase II trial will assess epcoritamab alone and in combination with lenalidomide as first-line treatment in older patients with DLBCL who are considered ineligible for anthracycline treatment (ClinicalTrials.gov Identifier: NCT05660967).

Comparison and validation of pre-, intra-, and post-treatment QoL assessments in older patients with DLBCL and the consideration of a combination of pre-and post-treatment measurements are needed. There is thus a need to establish evaluation methods for R-mini-CHOP/reduced-intensity R-CHOP alternatives in patients who cannot tolerate full-dose chemotherapy. A comparison of QoL outcomes between new alternative therapies is required, especially when clinical outcomes are similar.

Although the management of older patients with DLBCL continues to present a challenge, a new era has begun. Objective parameters defining patient fitness status provide the basis for establishing the correct treatment intensity and should be included in future clinical trials. Additionally, a QoL assessment and patient-reported outcomes should be considered as important endpoints. Novel agents with immunological mechanisms of action may help to improve outcomes for patients who have relapsed or become refractory after standard immunochemotherapy and those who are unable to receive standard immunochemotherapy due to comorbidities.

Treatment is not standardized for selected patients, particularly those >80 years old and frail patients, and reduced-intensity chemotherapy may achieve clinical outcomes comparable to full-dose chemotherapy in this population, while avoiding adverse effects. The selection of patients for reduced-intensity chemotherapy or chemotherapy-free regimens is predominantly undertaken using comprehensive or simplified frailty assessments based on evidence measuring clinical outcomes; however, baseline QoL and anticipated gains in QoL may also play important roles in selecting patients with DLBCL for individualized treatment among the older population.

## Figures and Tables

**Table 1:** First-line immunochemotherapy regimens for older patients with DLBCL.

Therapy Type	Age	Development Status	Reference(s)
R-CHOP	60–80 years	Phase 3 trial	Coiffier et al., 2002 [1]
R-mini-CHOP	≥80 years	Phase 2 trial	Peyrade et al., 2011 [28] Peyrade et al., 2017 [29]
Pola-R-CHP	<80 years	Phase 3 trial	Tilly et al., 2022 [5]
Pola-R-mini-CHP	≥80 years or frail patients aged ≥75 years	Phase 3 trial, ongoing	ClinicalTrials.gov Identifier: NCT04332822

Abbreviations: DLBCL, diffuse large B-cell lymphoma; R-CHOP, rituximab, cyclophosphamide, doxorubicin, vincristine, prednisone; R-mini-CHOP, attenuated-dose R-CHOP; Pola-R-CHP, of vincristine with polatuzumab vedotin (Pola), an antibody-drug-conjugated targeting CD79b, in the R-CHOP scheme.

**Table 2:** New, novel, and chemotherapy-free regimens.

Therapy Type	Example(s)	Development Status	Reference(s)
BTK inhibitor	Ibrutinib	Phase 2 trial	Xu et al., 2022 [63]
Tafa-Len	Tafa-Len	Phase 2 trial	Salles et al., 2020 [53] Duell et al., 2021 [54]
CD19 antibody–drug conjugate	Lonca	Phase 2 trial	Caimi et al., 2021 [55]
Bispecific antibody	Glofitamab Epcoritamab	Phase 2 trial	Dickinson et al., 2022 [64] Thieblemont et al., 2023 [65]
CAR T-cells	Axi-cel Liso-cel Tisa-cel	Phase 3 trial	Cocke et al., 2022 [68] Abramson et al., 2022 [69] Bishop et al., 2022 [70]

Abbreviations: BTK, Bruton’s tyrosine kinase; Tafa, tafasitamab; Len, lenalidomide; Lonca, loncastuximab; CAR, chimeric antigen receptor.

**Table 3:** Evaluation of frailty in older patients.

Assessment Tool	Key Parameters	Benefits	Limitations
Comprehensive geriatric assessment	ADL, IADL, CCI, CIRS-G	Recommended by ASCO (Mohile et al., 2018 [72])	Complexity, time (Moccia and Thieblemont, 2018 [17])
Simplified geriatric assessment (Merli et al., 2021 [75])	ADL, IADL, age, (≥80 years), CIRS-G, IPI, hemoglobin	OS, CR rate (Bai et al., 2020 [73])	Complexity
IADL (Lawton and Brody, 1969 [79])	IADL	Predicts OS with R-mini-CHOP therapy (Yamasaki et al., 2022 [78])	Reproducibility
G8 screening test (Martinez-Tapia et al., 2017 [80])	G8	OS, treatment toxicity	Reproducibility
Age, comorbidities, albumin index, and IADL (Hohloch et al., 2020 [83])	Age, CCI, albumin (≤3.5 g/dL)	OS, mean chemotherapy dose, treatment toxicity, treatment-related toxicity	Reproducibility
Vulnerable elders survey-13 (Ribi et al., 2015 [84])	Vulnerable elders survey-13	OS, response rate	Reproducibility
Prognostic nutritional index (Go et al., 2019 [85])	Prognostic nutritional index	OS	Reproducibility

Abbreviations: ADL, activities of daily living; CCI, Charlson comorbidity index; ASCO, American Society of Clinical Oncology; CIRS-G, Cumulative Illness Rating Scale for Geriatrics; IADL, Instrumental Activities of Daily Living; MCIRS-G, modified CIRS-G; OS, overall survival; IPI, international prognostic index.
